# An Electrochemical Sensor Based on Amino Magnetic Nanoparticle-Decorated Graphene for Detection of Cannabidiol

**DOI:** 10.3390/nano11092227

**Published:** 2021-08-29

**Authors:** Yi Zhang, Zongyi You, Chunsheng Hou, Liangliang Liu, Aiping Xiao

**Affiliations:** Institute of Bast Fiber Crops, Chinese Academy of Agricultural Sciences, Changsha 410205, China; zhang94721@gmail.com (Y.Z.); Xianyuxiaoyoujun@outlook.com (Z.Y.); houchensheng@caas.cn (C.H.)

**Keywords:** cannabidiol, *Cannabis sativa* L., electrochemical sensor, graphene, magnetic nanoparticles

## Abstract

For detection of cannabidiol (CBD)—an important ingredient in *Cannabis sativa* L.—amino magnetic nanoparticle-decorated graphene (Fe_3_O_4_-NH_2_-GN) was prepared in the form of nanocomposites, and then modified on a glassy carbon electrode (GCE), resulting in a novel electrochemical sensor (Fe_3_O_4_-NH_2_-GN/GCE). The applied Fe_3_O_4_-NH_2_ nanoparticles and GN exhibited typical structures and intended surface groups through characterizations via transmission electron microscopy (TEM), scanning electron microscopy (SEM), X-ray powder diffraction (XRD), vibrating sample magnetometer (VSM), and Raman spectroscopy. The Fe_3_O_4_-NH_2_-GN/GCE showed the maximum electrochemical signal for CBD during the comparison of fabricated components via the cyclic voltammetry method, and was systematically investigated in the composition and treatment of components, pH, scan rate, and quantitative analysis ability. Under optimal conditions, the Fe_3_O_4_-NH_2_-GN/GCE exhibited a good detection limit (0.04 μmol L^−1^) with a linear range of 0.1 μmol L^−1^ to 100 μmol L^−1^ (*r*^2^ = 0.984). In the detection of CBD in the extract of *C. sativa* leaves, the results of the electrochemical method using the Fe_3_O_4_-NH_2_-GN/GCE were in good agreement with those of the HPLC method. Based on these findings, the proposed sensor could be further developed for the portable and rapid detection of natural active compounds in the food, agricultural, and pharmaceutical fields.

## 1. Introduction

*Cannabis sativa* L. (*C. sativa*) is an annual dioecious herb belonging to the Cannabinaceae family, which is cultivated worldwide, and was one of the original crops in China [1]. *C. sativa* can be simply divided into industrial hemp and marijuana—generally distinguished by the content of Δ^9^-Tetrahydrocannabinol (Δ^9^-THC) in the plant; it is considered to be industrial hemp when the content of Δ^9^-THC is lower than 0.3% (w/w), and otherwise is referred to as marijuana. Based on the existing legal requirements in China, the cultivation of marijuana is banned; all varieties of *C. sativa* planted in China belong to the industrial hemp category [2]. In recent years, the medicinal usage of cannabidiol (CBD) has received unprecedented attention in the pharmaceutical and cosmetics industries. Accordingly, as the natural extraction source of CBD, *C. sativa* has ushered in a new round of development [3]. As an isomeride of Δ^9^-THC, CBD is non-psychoactive and exhibits good pharmacological effects in treating chronic pain, anxiety, inflammation, depression, and many other symptoms [4,5]. Today, the quantitative analysis methods of cannabinoids—including CBD—are mainly chromatographic methods, such as high-performance liquid chromatography (HPLC), gas chromatography (GC), and mass spectrometry [6,7,8].

As a kind of ultrasensitive detection method, electrochemical sensors or biosensors are mainly reported for the detection of Δ^9^-THC, since Δ^9^-THC is a typical psychoactive drug and is strictly regulated [9]. However, electrochemical sensors developed for the detection of CBD are rare. Since the interest in (and market for) CBD and related products are growing, the detection of CBD has also become more important [10]. A convenient and rapid strategy for the detection of CBD could meet many needs in various scenarios outside of the laboratory. Through customization in the size and composition of sensors, in combination with the design of a small and portable workstation, a preliminary and rapid detection of CBD in plants could be completed in the field, which could save a lot of work for farmers or researchers [11]. Therefore, the effort to develop electrochemical sensors for CBD using novel nanomaterials is worthwhile.

As a typical two-dimensional nanomaterial, graphene (GN) has displayed many properties, including large specific surface area, high chemical stability, and excellent electrochemical properties [12]. It is widely used to modify electrodes in order to achieve better results in applications of supercapacitors, potassium-ion batteries, and detectors for biomarkers, metabolites, viruses, etc. [13,14,15,16]. The beneficial effects of modification of electrochemical sensors have been proven, including route simplicity, high efficiency, good performance, and low cost [17]. Magnetic nanoparticles such as iron oxide also show characteristics such as low toxicity, ease of functionalization, high adsorption ability, and magnetic responsivity [18]. The introduction of magnetic nanoparticles in electrochemical sensors could facilitate of electron transfer and signal amplification. The combination of GN and magnetic nanoparticles, resulting in Fe_3_O_4_/GN nanocomposites, has been utilized in the construction of various electrochemical sensors [19]. The Fe_3_O_4_/GN nanocomposites have been applied with satisfactory performance in the electrochemical detection of arsenic ions, lobetyolin, dopamine, glucose, prostate-specific antigen, hepatitis C virus, etc. [20,21,22,23,24,25].

**Figure 1:**
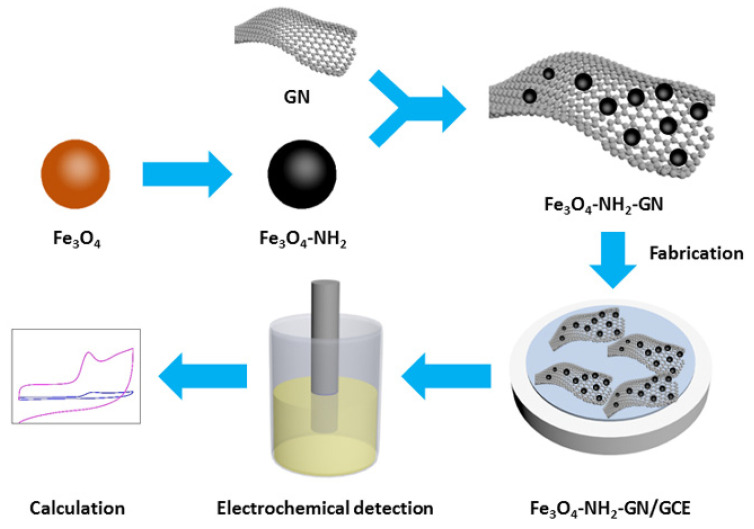
Diagram of Fe_3_O_4_-NH_2_-GN/GCE and electrochemical detection of CBD.

In this study, many materials were tested for the modification of electrodes in order to obtain higher signals in the electrochemical detection of CBD. Amino-group-modified Fe_3_O_4_ nanoparticles (Fe_3_O_4_-NH_2_) were finally confirmed for the modification of a glassy carbon electrode (GCE) together with GN in the form of nanocomposites. After the characterizations of the materials, GN and Fe_3_O_4_-NH_2_ were mixed as nanocomposites and modified on the GCE (Fe_3_O_4_-NH_2_-GN/GCE) to develop a novel electrochemical sensor for the highly selective and sensitive detection of CBD (Figure 1). The composition and fabrication sequences of the modifiers were investigated and optimized. Under the optimal fabrication and analytical conditions, the proposed Fe_3_O_4_-NH_2_-GN/GCE demonstrated enhanced electrochemical signals, good linearity, and satisfactory anti-interference ability for CBD. The CBD content of *C. sativa* leaf extract was detected using the proposed Fe_3_O_4_-NH_2_-GN/GCE, and the results were compared with those of the conventional HPLC method. Hence, it can be expected that the Fe_3_O_4_-NH_2_-GN/GCE has extensive potential applications in the detection of CBD and other natural ingredients.

## 2. Materials and Methods

### 2.1. Reagents and Apparatus

Detailed information about the reagents and instrumentations can be found in the Supplementary Material.

### 2.2. Preparation of Fe_3_O_4_-NH_2_ Nanoparticles

Fe_3_O_4_ nanoparticles were prepared according to our previous report [26]. Typically, 1.35 g of ferric chloride, 3.60 g of sodium acetate, and 1.00 g of PEG 6000 were mixed in 50 mL of ethylene glycol. The mixture was stirred under ultrasonication for 30 min and poured into a Teflon-lined stainless steel autoclave (100 mL). The autoclave was put into a drying oven at 180 °C for 6 h. After reaction, the black products were poured out and washed with water and ethanol three times each.

The obtained Fe_3_O_4_ nanoparticles were then dispersed in 250 mL of ethanol and ultrasonicated for 30 min [27]. After that, the materials were poured into a round-bottomed flask, and 2 mL of 3-aminopropyltriethoxysilane was dripped slowly into the Fe_3_O_4_ nanoparticle dispersion under mechanical agitation. The reaction was performed at room temperature for 6 h. Finally, the Fe_3_O_4_-NH_2_ nanoparticles were washed with ethanol three times and stored in ethanol at 4 °C.

### 2.3. Fabrication of the Fe_3_O_4_-NH_2_-GN/GCE

Before modification, the GCE was polished using alumina powders (0.05 μm) and cleaned via ultrasonication for 10 min. The surface of the GCE was dried with nitrogen gas and stored for further use. Next, 12.0 mg of Fe_3_O_4_-NH_2_ nanoparticles and 12.0 mg of GN were mixed in 2.0 mL of water and ultrasonicated for 10 min to form a homogeneous solution, which was marked as Fe_3_O_4_-NH_2_-GN nanocomposites. Then, 10 μL of the Fe_3_O_4_-NH_2_-GN suspension (6.0 mg/mL in water) was carefully dropped on the surface of GCE and air-dried to form an active layer on the surface of the electrode. The modified electrode was referred to as Fe_3_O_4_-NH_2_-GN/GCE.

For comparison, 10 μL of Fe_3_O_4_-NH_2_ nanoparticles and GN (6.0 mg/mL in water) were fabricated on the GCE in the same procedures and conditions, which were designated as Fe_3_O_4_-NH_2_/GCE and GN/GCE, respectively. For confirmation of the fabrication sequence, three kinds of sequences were compared. Fe_3_O_4_-NH_2_ nanoparticles were firstly dropped on the surface of GCE; GN was then dropped on the surface when the nanomaterials were dried, the result of which was designated as GN/Fe_3_O_4_-NH_2_/GCE. GN was firstly modified on the bare GCE, and then Fe_3_O_4_-NH_2_ nanoparticles were modified, which was designated as Fe_3_O_4_-NH_2_/GN/GCE. These two electrodes were compared with Fe_3_O_4_-NH_2_-GN/GCE for their electrochemical response under the same conditions.

### 2.4. Preparation of Real Sample

Dry *C. sativa* leaves were ground, passed through a 40-mesh sieve, and placed in an oven at 105 °C for 10 h. After these treatments, 0.5 g of *C. sativa* leaves was immersed in 50 mL of anhydrous methanol solution. The mixture was extracted for 20 min using an ultrasonic extractor at a power of 200 W (KQ5200DV, Kunshan Ultrasonic Instrument Co., Ltd., Kunshan, China). After extraction, the mixture was centrifuged at 4000 r/min for 5 min (TD5, Yingtai Instrument Co., Ltd., Changsha, China). Next, 2 mL of the upper transparent solution was diluted to 20 mL with phosphate buffer solution (PBs, 10 mmol L^−1^, pH 5.0) and filtered with 0.45 μm filter before analyses via HPLC and using the proposed sensor.

### 2.5. Determination of CBD by HPLC

For comparison of the detection results, the HPLC method was applied in the detection of samples as well. An isocratic elution program consisting of 0.1% acetic acid and 75% acetonitrile was applied for 30 min at 25 °C. The flow rate was set to 0.8 mL/min. The chromatogram was observed at 220 nm. The injection volume of the sample was 10 μL. The CBD content in the *C. sativa* leaf extract was calculated using the standard curve obtained by the measurement standards.

### 2.6. Electrochemical Measurements

The electrochemical measurements were performed using the three-electrode system in CBD solution, using PBs (10 mmol L^−1^, pH 5.0, containing 10% methanol) as a solvent and supporting electrolyte. Cyclic voltammetry (CV) was used for the measurement, with a scan rate of 0.05 V s^−1^ and a potential range from 0 V to 0.8 V. Electrochemical impedance spectroscopy (EIS) was applied to characterize the sensor conductivity in the solution containing 5.0 mmol L^−1^ of K_3_[Fe(CN)_6_]/K_4_[Fe(CN)_6_] and 0.1 mol L^−1^ of potassium chloride. The amplitude was 0.005 V with a frequency range of 0.1 to 10^5^ Hz. All experiments were carried out in three duplicates at 25 ± 2 °C.

## 3. Results and Discussion

### 3.1. Characterizations of Nanomaterials

#### 3.1.1. TEM and SEM

**Figure 2:**
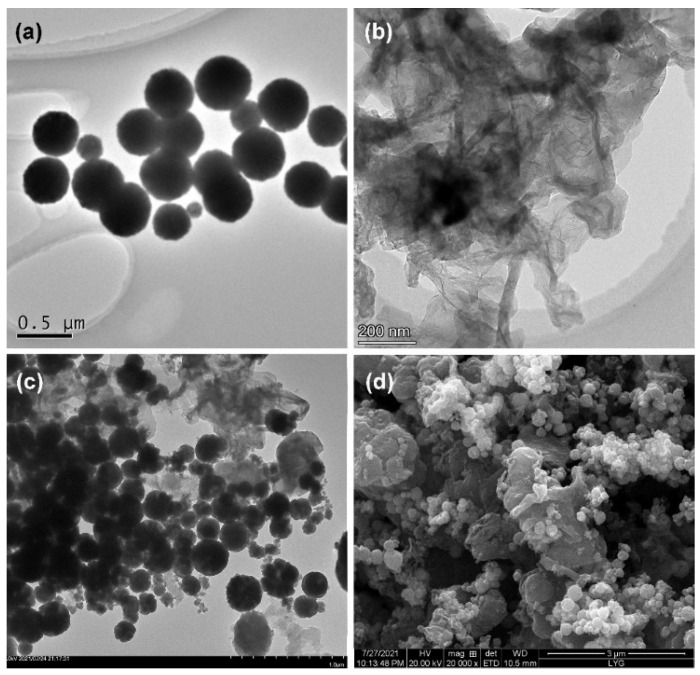
TEM images of (**a**) Fe_3_O_4_, (**b**) GN, and (**c**) Fe_3_O_4_-NH_2_-GN. (**d**) SEM image of Fe_3_O_4_-NH_2_-GN.

The morphologies of Fe_3_O_4_ nanoparticles, GN, and Fe_3_O_4_-NH_2_-GN were investigated via TEM (Figure 2). The round sphere of Fe_3_O_4_ nanoparticles, as well as the silk-like and wrinkled structures of GN, could be easily observed in the corresponding images (Figure 2a,b) [28]. The Fe_3_O_4_ nanoparticles showed sizes of about 430 nm, with good dispersion. After mixing, the Fe_3_O_4_-NH_2_-GN nanocomposites retained the characteristics of Fe_3_O_4_ nanoparticles and GN. It can be seen in the TEM image that Fe_3_O_4_ nanoparticles were dispersed on the GN sheets (Figure 2c) [29].

An SEM image of the Fe_3_O_4_-NH_2_-GN nanocomposites on the electrode surface was also provided in order to confirm the morphology and structure (Figure 2d), and showed the modified surface of the electrode. Though there was a kind of agglomeration in the nanocomposites, the existence of Fe_3_O_4_ nanoparticles (round spheres) on the GN could be confirmed. The irregular surface of the modified electrode might be one of the reasons for the improved electrochemical response.

#### 3.1.2. XRD

**Figure 3:**
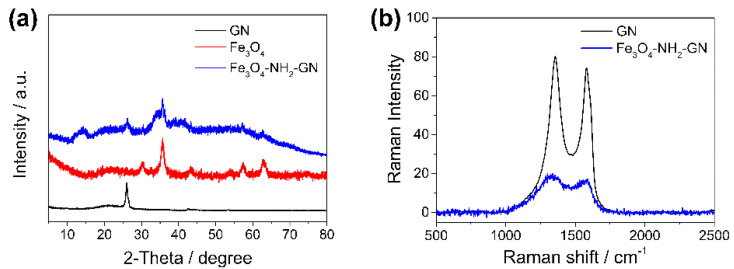
(**a**) XRD patterns of GN, Fe_3_O_4_, and Fe_3_O_4_-NH_2_-GN. (**b**) Raman spectra of GN and Fe_3_O_4_-NH_2_-GN.

The XRD patterns of Fe_3_O_4_ nanoparticles, GN, and Fe_3_O_4_-NH_2_-GN were analyzed, and are shown in Figure 3a. The pattern of Fe_3_O_4_ nanoparticles exhibited typical peaks at 30.3°, 35.7°, 43.6°, 57.4°, and 62.9, which were attributed to the indices (220), (311), (400), (511), and (440) of the Fe_3_O_4_ crystal, respectively [30]. Meanwhile, in the pattern of Fe_3_O_4_-NH_2_-GN, the related peaks became much weaker, which might be a result of the coating of GN and the modification of the amino groups [26]. Additionally, another peak at ~26° could be observed, belonging to the characteristic reflection of the existence of GN [31]. The XRD results confirmed the existence of Fe_3_O_4_ nanoparticles and GN in the Fe_3_O_4_-NH_2_-GN nanocomposites.

#### 3.1.3. Raman

Figure 3b illustrates the Raman spectra of the GN and Fe_3_O_4_-NH_2_-GN nanocomposites. Both GN and Fe_3_O_4_-NH_2_-GN showed two peaks at around 1350 cm^−1^ and 1570 cm^−1^, which were designated as D band and G band; they represented the disordered sp3 carbon structure (D band) and the sp2 ordered crystalline structure (G band) of GN [32]. After the combination of the two nanomaterials, the intensities of the peaks reduced significantly, which might be a result of the introduction of Fe_3_O_4_-NH_2_ nanoparticles. However, the intensity ratio of the D to G peaks was maintained, showing that the structure of GN was not affected.

### 3.2. Electrochemical Characteristics

**Figure 4:**
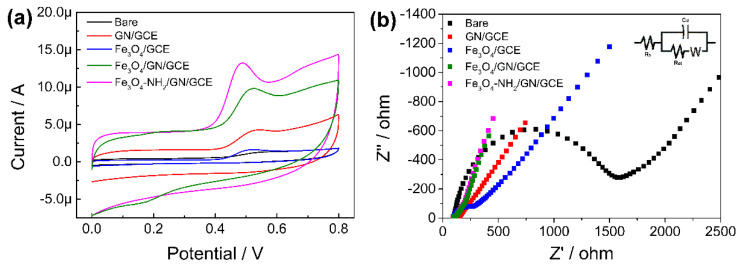
(**a**) CV curves of CBD on bare GCE, GN/GCE, Fe_3_O_4_/GCE, Fe_3_O_4_-GN/GCE, and Fe_3_O_4_-NH_2_-GN/GCE. CV method: 100 μmol L^−1^ of CBD in 10 mmol L^−1^ of PBs (pH 5.0, containing 10% methanol). Potential range of 0–0.8 V. Scan rate of 0.05 V s^−1^. (**b**) Nyquist plots of bare GCE, GN/GCE, Fe_3_O_4_/GCE, Fe_3_O_4_-GN/GCE, and Fe_3_O_4_-NH_2_-GN/GCE in 5.0 mmol L^−1^ of K_3_[Fe(CN)_6_]/K_4_[Fe(CN)_6_] and 0.1 mol L^−1^ of potassium chloride. The amplitude is 0.005 V, with a frequency range of 0.1 to 10^5^ Hz.

The electrochemical behavior of various modified electrodes in 100 μmol L^−1^ of CBD were compared via the CV method. As shown in Figure 4a, the electrochemical response of CBD on bare GCE was only 0.728 μA (black line). After the respective modifications with Fe_3_O_4_ nanoparticles and GN to the GCE, small oxidation peaks at around 0.5 V could be observed on the Fe_3_O_4_/GCE (blue line) and the GN/GCE (red line), which might be due to the electron transfer properties and the good conductivity of Fe_3_O_4_ nanomaterials and GN [33]. When Fe_3_O_4_-GN suspensions were used to modify the GCE, resulting in the Fe_3_O_4_-GN/GCE, an apparent increase in peak current could be observed (5.659 μA, green line). The advantages of GN and Fe_3_O_4_ nanoparticles were combined and enhanced. Moreover, when the Fe_3_O_4_ nanoparticles were functionalized by amino groups, the resulting modified electrode (Fe_3_O_4_-NH_2_-GN/GCE) showed the highest response among these electrodes (8.978 μA, Pink line). In order to confirm the effect of amino groups on Fe_3_O_4_ nanoparticles, Fe_3_O_4_-nanoparticle- and Fe_3_O_4_-NH_2_-nanoparticle-modified electrodes (Fe_3_O_4_/GCE and Fe_3_O_4_-NH_2_/GCE) were compared. As a result, the peak current of Fe_3_O_4_-NH_2_/GCE was slightly higher than that of Fe_3_O_4_/GCE (1.366 to 1.08, not shown). A possible reason for this increase might be that the amino groups on the surface could attract more target molecules. As far as we know, there has been no previous report regarding the electrochemical oxidation mechanism of CBD. By referring to reported works on the oxidation of Δ^9^-THC, the oxidation process of CBD could be assumed to be a phenol-type oxidation mechanism [34,35].

In order to optimize the effects of the modifiers, the fabrication sequence of modified sensors was investigated. Through the comparison of GN/Fe_3_O_4_-NH_2_/GCE, Fe_3_O_4_-NH_2_/GN/GCE, and Fe_3_O_4_-NH_2_-GN/GCE, the peak currents of each sensor were obtained, as shown in Table 1. Apparently, the Fe_3_O_4_-NH_2_-GN/GCE showed the best response among these sensors, meaning that the modifiers should first be mixed, and then dropped directly on the surface of the electrode. Based on this finding, different preparation methods of Fe_3_O_4_-NH_2_-GN suspensions were tried (see the ESM). Three kinds of Fe_3_O_4_-NH_2_-GN nanocomposites were compared, and their corresponding peak currents are also shown in Table 1. Although the Fe_3_O_4_ nanoparticles were directly prepared in the presence of GN via ultrasonication and solvothermal methods, the electrochemical properties obtained were not as good as via the physical mix method. Hence, the Fe_3_O_4_-NH_2_-GN suspension was confirmed as the optimal material in this research.

The Nyquist plots from the EIS test reflect the conductivity of the electrodes (Figure 4b). The inset of Figure 4b shows a general equivalent circuit containing the solution resistance (R_s_), the electron transfer resistance (R_et_), the Warburg element (W), and the charge of the constant phase element (C_d_) [36]. The value of R_et_ was calculated by fitting the experimental data to the model circuit. As shown in Figure 4b, the Nyquist plot of bare GCE showed a semicircle, with an R_et_ of 1287 Ω. When the GCE was modified with Fe_3_O_4_ nanoparticles and GN, the R_et_ of Fe_3_O_4_/GCE and GN/GCE reduced to 141.4 Ω and 28.61 Ω, respectively. Finally, the R_et_ of Fe_3_O_4_-NH_2_-GN/GCE was only 13.73 Ω, which was similar to that of Fe_3_O_4_-GN/GCE (16.19 Ω). The decreases in resistance could be attributed to the outstanding electric conductivity of GN and magnetic nanoparticles [37]. Consequently, the Fe_3_O_4_-NH_2_-GN/GCE was confirmed as the optimal modified sensor, by reason of its optimal response and conductivity in electrochemical detection.

### 3.3. Optimization of Electrochemical Conditions

#### 3.3.1. Effect of Composition of Fe_3_O_4_-NH_2_-GN

**Figure 5:**
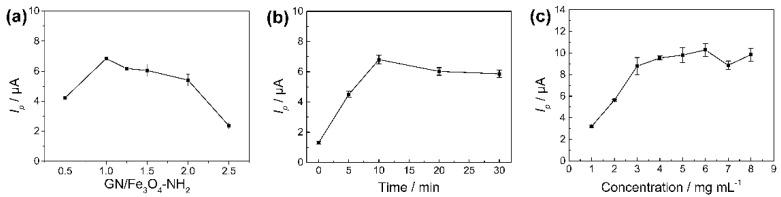
(**a**) Effect of compositions of Fe_3_O_4_-NH_2_-GN on *I_p_* in CV. (**b**) Effect of ultrasonication time of Fe_3_O_4_-NH_2_-GN on *I_p_* in CV. (**c**) Effect of modification volumes on peak current in CV. CV method: 100 μmol L^−1^ of CBD in 10 mmol L^−1^ of PBs (pH 5.0, containing 10% methanol). Potential range of 0–0.8 V. Scan rate of 0.05 V s^−1^.

In order to obtain the optimal mixture composition, the ratios of GN and Fe_3_O_4_-NH_2_ nanoparticles (1:0.5, 1:1, 1:1.25, 1:1.5, 1:2.0 and 1:2.5, w:w) were investigated, and are shown in Figure 5a. The concentration of GN was set at 2.0 mg mL^−1^, and the concentrations of Fe_3_O_4_-NH_2_ nanoparticles were verified according to the ratios. These illustrated results suggested that the electrochemical signals of CBD were the highest when the ratio was 1:1. When the ratio of Fe_3_O_4_-NH_2_ nanoparticles was higher than 1.0, the response gradually became weaker. Therefore, the material ratio of GN and Fe_3_O_4_-NH_2_ nanoparticles was set to 1:1 as the optimal composition for the fabrication of the electrode.

#### 3.3.2. Effect of Ultrasonication Time of Fe_3_O_4_-NH_2_-GN

To obtain a stable dispersion, various ultrasonication times of the Fe_3_O_4_-NH_2_-GN suspension were tested, from 1 min to 30 min (1, 5, 10, 20, and 30 min). Then, the materials were used for the fabrication of electrodes. The changes in peak currents using the corresponding modified electrodes are plotted in Figure 5b. It can be seen that the electrochemical response was the highest when the material was treated for 10 min. However, longer ultrasonication time did not make the response better. Hence, the ultrasonication time of Fe_3_O_4_-NH_2_-GN suspension was confirmed at 10 min.

#### 3.3.3. Effect of Concentration of Fe_3_O_4_-NH_2_-GN

The effect of concentration of Fe_3_O_4_-NH_2_-GN suspension was measured from 1.0 mg mL^−1^ to 8.0 mg mL^−1^, and the modification volume was fixed at 10.0 μL. As the previous experiment indicated, the ratio of GN and Fe_3_O_4_-NH_2_ nanoparticles was set at 1:1 (w:w). It can be seen in Figure 5c that the peak current increased as the concentration increased from 1.0 mg mL^−1^ to 6.0 mg mL^−1^. However, when the concentrations were more than 6.0 mg mL^−1^, this trend stopped, and the response began to gradually drop, which was similar to the results of a previous report [38]. Then, the concentration of Fe_3_O_4_-NH_2_-GN suspension was optimized as 6.0 mg mL^−1^.

#### 3.3.4. Effect of pH

**Figure 6:**
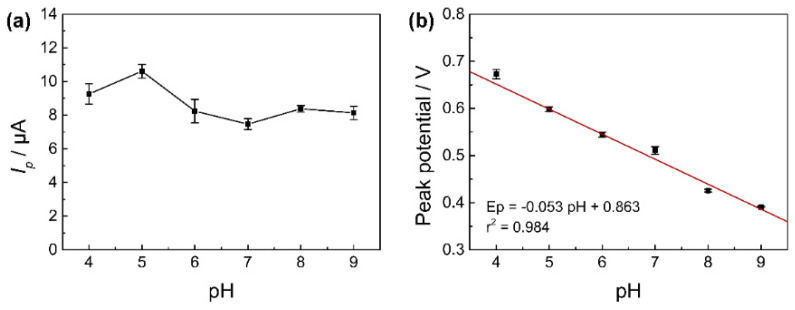
(**a**) Effect of pH on *I_p_* in CV. (**b**) Plot of peak potential (Ep) to pH values. CV method: 100 μmol L^−1^ of CBD in 10 mmol L^−1^ of PBs (pH 5.0, containing 10% methanol). Potential range of 0−0.8 V. Scan rate of 0.05 V s^−1^.

The electrochemical detection using various pH values of the electrolyte (4.0, 5.0, 6.0, 7.0, 8.0, and 9.0) containing CBD as samples was performed with the Fe_3_O_4_-NH_2_-GN/GCE, using the CV method. The trend is shown in Figure 6a, and the peak current of CBD was the highest when the pH of the electrolyte was 5.0. There was a downward trend when the pH of the electrolyte became higher than 5.0, which is consistent with Zanardi’s research [39]. Thus, 5.0 was adopted as the optimal electrolyte pH value during the tests.

Moreover, it could be observed that there was a linear shit of the peak potential (Ep) to lower positive values as the pH increased. The linear equation between Ep and pH was expressed as Ep = −0.053 pH + 0.863 (*r*^2^ = 0.984) (Figure 6b). The slope of the equation was ~−0.053 V pH^−1^, similar to the theoretical Nernstian slope of 0.059 V pH^−1^. This parameter corresponded to an oxidation mechanism that included the exchange of an equal number of protons and electrons in the reaction [40].

### 3.4. The Influence of the Scan Rate

**Figure 7:**
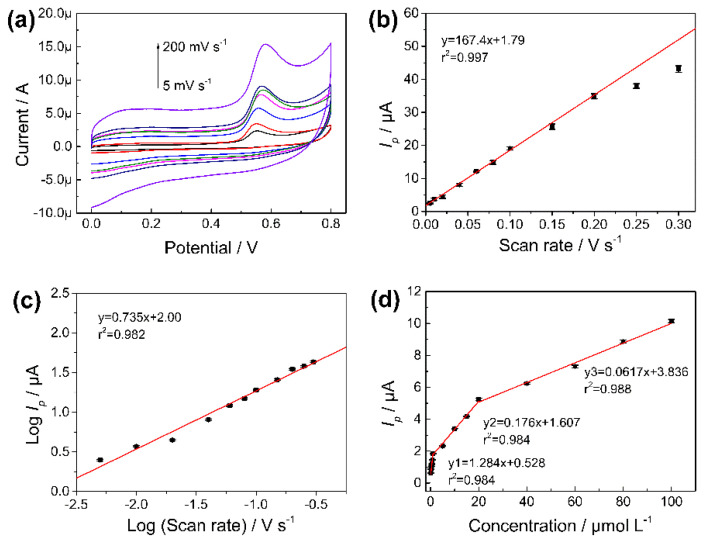
(**a**) CV of Fe_3_O_4_-NH_2_-GN/GCE in CBD solution at different scan rates. (**b**) The linear graph of *I_p_* and scan rates. (**c**) The linear graph of log *I_p_* and log (scan rate). (**d**) Plot of *I_p_* versus concentration of CBD. CV method: 100 μmol L^−1^ of CBD in 10 mmol L^−1^ of PBs (pH 5.0, containing 10% methanol). Potential range of 0–0.8 V. Scan rate of 0.05 V s^−1^.

As an important parameter reflecting the performance of the electrode, the effect of different scan rates (from 5 mV s^−1^ to 200 mV s^−1^) on the electrochemical response of CBD in PBs (10 mmol L^−1^, pH 7.0) was evaluated on the Fe_3_O_4_-NH_2_-GN/GCE, using the CV method. Figure 7a shows the resulting CV curves at a variety of scan rates. It can be seen that the peak currents increased and shifted with the increasing scan rates. A good linear relationship could be obtained between scan rate and peak current, which could be expressed as: *I_p_* = 164.84 *v* + 1.73 (*r*^2^ = 0.998) (Figure 7b), indicating that the oxidation of CBD was an adsorption-controlled process [41]. However, when the scan rate was increased to more than 200 mV s^−1^ (250 mV s^−1^ and 300 mV s^−1^), the response did not grow proportionately to the former linear trend (lower than former trend). Another linear dependence of the logarithm of the peak current (log *I_p_*) against the logarithm of the scan rate could also be observed, which was fitted as: log *I*_p_ = 0.735 log *v* + 2.00 (*r*^2^ = 0.982) (Figure 7c). This trend suggests that the electrochemical reaction was controlled by both diffusion and adsorption [42].

### 3.5. Quantitative Analysis of CBD

In order to study the quantitative analysis ability of the fabricated Fe_3_O_4_-NH_2_-GN/GCE, the CV curves of CBD at different concentrations from 0.1 μmol L^−1^ to 100 μmol L^−1^ were observed in PBs (0.01 mol/L, pH 5.0). The results illustrated that the peak current increased with increasing CBD concentrations, and three sections of linear dependences could be found between the peak current and the CBD concentration during this range, with a detection limit of 0.04 μmol L^−1^ (S/N = 3), which is consistent with Liu’s work [43]. The plot of peak current versus CBD concentration is shown in Figure 7d. The three regression equations could be respectively expressed as: *I_p1_* = 1.284 C_1_ + 0.528 (0.1–0.974 μmol L^−1^, *r*^2^ = 0.984), *I_p2_* = 0.176 C_2_ + 1.607 (0.974–19.494 μmol L^−1^, *r*^2^ = 0.984), and *I_p3_* = 0.0617 C_3_ + 3.836 (19.494–100 μmol L^−1^, *r*^2^ = 0.988). It could be found in three regression equations that the slope of the peak current at low concentration was higher than at high concentration. At a lower analyte concentration, the number of active sites on the electrode was relatively higher. However, because of the occupancy of—and decrease in the number of—active sites at higher analyte concentrations, the sensitivity and the slope became lower [44]. This demonstrates that the quantitative analysis of CBD using the Fe_3_O_4_-NH_2_-GN/GCE was interesting and acceptable [45]. The detection abilities of the reported electrochemical sensors for CBD are listed and compared with the Fe_3_O_4_-NH_2_-GN/GCE in Table 2. Through the comparison, the proposed Fe_3_O_4_-NH_2_-GN/GCE exhibited a competitive detection capability and sensitivity for CBD.

### 3.6. Practicability of the Fe_3_O_4_-NH_2_-GN/GCE

The anti-interference ability, repeatability, and stability of the Fe_3_O_4_-NH_2_-GN/GCE were tested, and the results were satisfactory (see the ESM). Moreover, the detection ability of the Fe_3_O_4_-NH_2_-GN/GCE for CBD was evaluated in the extract of *C. sativa* leaves. In order to verify the results, the standard addition method was employed by spiking different amounts of CBD into samples. The results are shown in Table 3 and compared with those obtained via the HPLC method. The recoveries ranged from 99.1% to 100.4%, indicating that the determination was reliable, and there was consistency between the concentrations of CBD measured by both electrochemical and HPLC methods.

## 4. Conclusions

In this study, an electrochemical sensor (Fe_3_O_4_-NH_2_-GN/GCE) was fabricated for the detection of CBD. The applied materials and fabrication conditions were compared and optimized via various characterizations and evaluations. The performance of the Fe_3_O_4_-NH_2_-GN/GCE was investigated for aspects including pH, scan rate, anti-interference ability, repeatability, and stability. As a result, the proposed Fe_3_O_4_-NH_2_-GN/GCE showed an improved electrochemical response compared to a bare GCE. It displayed quantitative analysis ability for CBD, with a linear range of 0.1 μmol L^−1^ to 100 μmol L^−^^1^. The practicability test also showed that the result was in good agreement with that of the HPLC method in the detection of CBD in real samples. Based on these findings, the Fe_3_O_4_-NH_2_-GN/GCE could be further utilized for the detection of active compounds in natural extracts.

## Figures and Tables

**Table 1:** The *I_p_* in different fabrication sequences and process methods of Fe_3_O_4_-NH_2_-GN in modified electrodes.

Fabrication Sequence	*I_p_* (μA)	Process Method	*I_p_* (μA)
GN/Fe_3_O_4_-NH_2_/GCE	1.808	Ultrasonication	3.352
Fe_3_O_4_-NH_2_/GN/GCE	3.388	Solvothermal	4.232
Fe_3_O_4_-NH_2_-GN/GCE	5.327	Mix	5.550

**Table 2:** Comparison of different reported sensors for the electrochemical determination of CBD.

Electrode	Linear Rage (μmol L^−1^)	LOD (μmol L^−1^)	Ref.
GC/CB	0.96–6.37	0.35	[39]
Sonogel-Carbon-PEDOT	1.59–19.1	0.94	[34]
NACE–ED	0.32–31.8	0.064	[46]
GCE	-^NM^	-^NM^	[47]
Fe_3_O_4_-NH_2_-GN/GCE	0.1–100.0	0.04	This study

GC/CB: glassy carbon/carbon black; PEDOT: poly-(3,4-ethylenedioxythiophene); NACE–ED: non-aqueous capillary electrophoresis–electrochemical detection; ^NM^: Not mentioned.

**Table 3:** Determination of CBD in real samples. (*n* = 3).

Samples	Added (μmol L^−1^)	Found (μmol L^−1^)	Recovery (%)	RSD (%)	HPLC (μmol L^−1^)
Extract of *C. sativa*	0	11.95	-	2.23	12.06
	1.0	13.00	100.4	2.05	-
	5.0	16.84	99.1	3.51	-

## Data Availability

The data presented in this study are available on request from the corresponding author.

## Supplementary Materials

The following are available online at https://www.mdpi.com/article/10.3390/nano11092227/s1, Figure S1: (a) *I_p_* ratios of Fe_3_O_4_-NH_2_-GN/GCE in CBD solution containing various interfering substances. CV method: 100 μmol L^−1^ of CBD in 10 mmol L^−1^ of PBs (pH 5.0, containing 10% methanol). Potential range at 0–0.8 V. Scan rate at 0.05 V s^−1^, Figure S2: Repeatability of Fe_3_O_4_-NH_2_-GN/GCE in CBD solution. CV method: 100 μmol L^−1^ of CBD in 10 mmol L^−1^ of PBs (pH 5.0, containing 10% methanol). Potential range at 0–0.8 V. Scan rate at 0.05 V s^−1^, Figure S3: Stability of Fe_3_O_4_-NH_2_-GN/GCE in CBD solution. CV method: 100 μmol L^−1^ of CBD in 10 mmol L^−1^ of PBs (pH 5.0, containing 10% methanol). Potential range at 0–0.8 V. Scan rate at 0.05 V s^−1^.
