# Supplementary material for: An Electrochemical Sensor Based on Amino Magnetic Nanoparticle-Decorated Graphene for Detection of Cannabidiol
Source: Nanomaterials (Basel). 2021 Aug 29;11(9):2227. doi: 10.3390/nano11092227 (PMC8467804; doi:10.3390/nano11092227)
Supplement: Supplementary file 1 [file nanomaterials-11-02227-s001.zip › nanomaterials-1342669-supplementary.pdf]

nanomaterials

Article

An Electrochemical Sensor Based on Amino Magnetic Nanoparticle-Decorated Graphene for Detection of Cannabidiol

Yi Zhang, Zongyi You, Chunsheng Hou, Liangliang Liu * and Aiping Xiao *

Institute of Bast Fiber Crops, Chinese Academy of Agricultural Sciences, Changsha 410205, China; zhang94721@gmail.com (Y.Z.); Xianyuxiaoyoujun@outlook.com (Z.Y.); houchensheng@caas.cn (C.H.)

* Correspondence: liuliangliang@caas.cn (L.L.); aipingxiao@yahoo.com (A.X.); Tel.: +86-731-88998525 (L.L.), +86-731-88998536 (A.X.)

1. Experimental

1.1. Reagents and Materials

Graphene (GN) was purchased from Nanjing XFNANO Materials Tech Co., Ltd. (Nanjing, China). Disodium hydrogen phosphate, sodium dihydrogen phosphate, potassium chloride, sodium acetate, ferric chloride, polyethylene glycol (PEG) 6000, 3-aminopropyltriethoxysilane and ethylene glycol were obtained from Sinopharm Chemical Reagent Co., Ltd. (Shanghai, China). Acetonitrile and acetic acid in chromatographic grade were obtained from Macklin Inc. (Shanghai, China). Cannabidiol (CBD, 99.0 %), Δ^9-Tetrahydrocannabinol (THC, 99.0 %), Cannabidiolic acid (CBDA, 99.0 %) were purchased from Cerilliant as standards (Round Rock, Texas, USA). Deionized water used in experiments was supplied by an ELGA water purification system (ELGA Berkefeld, Veolia, Germany). *C. sativa* leaves were collected from the experimental base belonging to our research institute, Chinese academy of agricultural sciences. All other chemicals were bought from Sinopharm Chemical Reagent Co., Ltd. (Shanghai, China) with analytical grade.

1.2. Instrumentation

The electrochemical measurements were conducted using a CHI 660E electrochemical workstation (Shanghai Chenhua Co., Ltd., China). The experiments were carried out using a three-electrode system including the Ag/AgCl electrode as the reference, the platinum wire electrode as the auxiliary electrode and the glassy carbon electrode with a diameter of 3 mm as the working electrode (GaossUnion Technology Co. Ltd., Wuhan, China). The working electrode would be modified with various nanomaterials in this study.

The morphology of materials was observed by transmission electron microscope (TEM) on a Hitachi HT7700 transmission electron microscopy (Hitachi High Tech Co. Ltd, Tokyo, Japan), and scanning electron microscopy (SEM) on a JSM 6610Lv scanning electron microscope (JEOL, Tokyo, Japan). The structure analysis of materials was performed by X-ray powder diffraction (XRD) on a RINT 2500 powder X-ray Diffractometer using Cu-K(α) radiation (Rigaku Corporation, Tokyo, Japan). The magnetic properties of materials were obtained by a vibration sample magnetometer VSM7407 (Lake Shore, Westerville, OH, USA). The Raman spectra were measured by a Renishaw Invia Raman spectrometer (Wotton-under-Edge, UK).

HPLC analysis was completed on the Agilent 1260 HPLC included a quaternary pump, an autosampler, a thermostatic column compartment and a diode array detector (Agilent Technologies Inc., Santa Clara, CA, USA). An Agilent ZORBAX C_{18} column was employed (250 mm× 4.6 mm i.d.; 5 μm, Santa Clara, CA, USA).

1.3. Preparation of F_3O_4*-GN Nanocomposites*

Ultrasonication method [1]: 0.035 g of ferrous sulfate, 0.1 g of ferric chloride and 30 mg of GN were mixed in 20 ml of ethanol solution (50%, v:v) under vigorous stirring and ultrasonication. Under the ultrasonication, some aqueous ammonia was dropped into the mixture to make the pH value reached 10. The black nanocomposites could be magnetic separated and washed with ethanol.

Solvothermal method: Fe_3O_4 nanoparticles were prepared in the presence of GN to make the nanocomposites. 0.13 g of ferric chloride, 0.36 g of sodium acetate, 0.10 g of PEG 6000 and 30 mg of GN were mixed in 10 mL of ethylene glycol. The mixture was stirred under ultrasonication for 30 min and poured in a Teflon-lined stainless-steel autoclave (50 mL). The autoclave was put into a drying oven at 180 °C for 6 h. After reaction, the products were magnetic separated and washed with ethanol three times.

2. Results and Discussion

The anti-interference ability of Fe_3O_4-NH_2-GN/GCE toward inorganic metal ions were studied. The concentrations of these interfering inorganic metal ions were 50-fold higher than that of CBD (5.0 mmol L^{-1} to 100 μmol L^{-1}). The I_p changed ratios (%) were shown in **Figure S1** in the respective additions of Na^+, K^+, Fe^{3+}, Ca^{2+} and Al^{3+} ions. The I_p changed ratios of interfering ions were ranged from 2.5% to 6.8%.

The reproducibility of Fe_3O_4-NH_2-GN/GCE in detection was studied by consecutive detections for six times using the same sensor. During these six tests, the fluctuation of peak currents was acceptable with the relative standard deviation (RSD) value at 1.4% (**Figure S2**), which showed an admissible precision of this fabricated sensor.

The stability of Fe_3O_4-NH_2-GN/GCE was also measured by monitoring the responses for the same concentrations of CBD on six days. The peak currents remained at 93.0% of the initial value with RSD of 2.4% (**Figure S3**). These results showed the Fe_3O_4-NH_2-GN/GCE had a satisfactory anti-interference ability, reproducibility, and stability for detection of CBD.

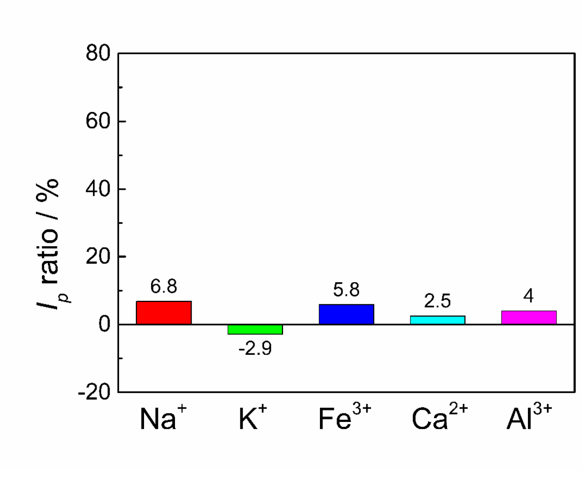

Figure S1. I_p ratios of Fe_3O_4-NH_2-GN/GCE in CBD solution containing various interfering substances. CV method: 100 μmol L^{-1} of CBD in 10 mmol L^{-1} of PBs (pH 5.0, containing 10% methanol). Potential range at 0–0.8 V. Scan rate at 0.05 V s^{-1}.

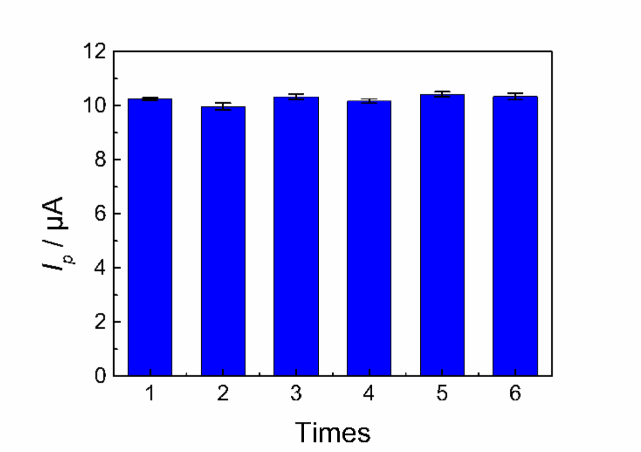

Figure S2. Reproducibility of Fe_3O_4-NH_2-GN/GCE in CBD solution. CV method: 100 μmol L^{-1} of CBD in 10 mmol L^{-1} of PBs (pH 5.0, containing 10% methanol). Potential range at 0–0.8 V. Scan rate at 0.05 V s^{-1}.

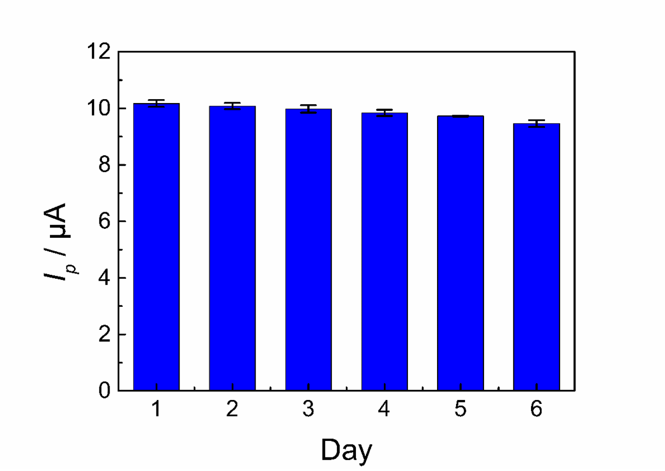

Figure S3. Stability of Fe_3O_4-NH_2-GN/GCE in CBD solution. CV method: 100 μmol L^{-1} of CBD in 10 mmol L^{-1} of PBs (pH 5.0, containing 10% methanol). Potential range at 0–0.8 V. Scan rate at 0.05 V s^{-1}.

Reference

1. Suhanto, R.N.; Harimurti, S.; Septiani, N.L.W.; Utari, L.; Anshori, I.; Wasisto, H.S.; Suzuki, H.; Suyatman; Yuliarto, B., Sonochemical synthesis of magnetic Fe_3O_4/graphene nanocomposites for label-free electrochemical biosensors. *J. Mater. Sci.-Mater. Electron.* **2020,** *31*, 15381–15393.
